# The use of flexible laryngeal mask airway for Adenoidectomies: An experience of 814Paediatric patients

**DOI:** 10.12669/pjms.334.12432

**Published:** 2017

**Authors:** Ozlem Ozmete, Mesut Sener, Esra Caliskan, Meltem Kipri, Anis Aribogan

**Affiliations:** 1 Ozlem Ozmete, MD, Department of Anesthesiology and Reanimation, Baskent University School of Medicine, Adana, Turkey; 2 Prof. MesutSener, MD, Department of Anesthesiology and Reanimation, Baskent University School of Medicine, Adana, Turkey; 3 Asocc. Prof. Esra Caliskan, MD, Department of Anesthesiology and Reanimation, Baskent University School of Medicine, Adana, Turkey; 4 Meltem Kipri, MD, Department of Anesthesiology and Reanimation, Baskent University School of Medicine, Adana, Turkey; 5 Prof. Anis Aribogan, MD, Department of Anesthesiology and Reanimation, Baskent University School of Medicine, Adana, Turkey

**Keywords:** Adenoidectomy, Children, Flexible-laryngeal mask airway, Peroperative complications

## Abstract

**Objective:**

To assess flexible laryngeal mask airway (F-LMA) use during pediatric adenoidectomies in terms of patient safety, comfort, complication rates and surgeon satisfaction levels.

**Methods:**

Patients who had undergone an elective adenoidectomy after receiving general anesthesia using F-LMA from June 2012 to November 2015 were included. Patients’ demographics and the incidence of perioperative complications were investigated. The surgeon’s satisfaction level was also evaluated by questionnaire.

**Results:**

Eight hundred fourteen patient were included in the study. Conversion from F-LMA to an endotracheal tube was carried out in two patients (0.2%). Airway complications were identified in two patients. The mean duration of stay in the postoperative anesthesia care unit was 17 minutes. All patients were discharged the same day. According to the otolaryngologists F-LMA applications provide a significant reduction in the processing time (100%), postoperative patient comfort is better than when using endotracheal intubation (83.3%) and the consensus was that there should be a complete continuation of the use of the F-LMA (100%) in subsequent adenoidectomies.

**Conclusion:**

Our data show that the use of F-LMA for pediatric adenoidectomies has well tolerability profile and resulted in a lower incidence of complications. We think that the use of F-LMA for pediatric adenoidectomy is safer, simpler and speeder method.

## INTRODUCTION

Adenoidectomies are most widely performed surgical operation in the childhood. In anesthesiology practice, endotracheal intubation (ETI) is considered the standard method for airway management, especially during operations where that airway is used with surgeons.

The use of general anesthesia with ETI is widely preferred in children undergoing adenoidectomy. Recently, it has been shown that a safe airway can be achieved using a laryngeal mask airway (LMA) in children undergoing adenoidectomy.^1^-^3^ Studies have indicated that, in addition to ensuring airway safety, LMA application does not involve the use of muscle relaxant agents, does not cause laryngospasm, bronchospasm, or breathe holding, and allows for early intubation. Therefore, LMA application was found to be superior to or at least not inferior to ETI with respect to respiratory complications.^1^-^3^ However, there is still debate on the safe airway management of adenotonsillectomies in terms of perioperative airway complications when using a classic LMA such as the displacement of the LMA, gas leaking when positioning for surgery or a kink during the operation.^4^,^5^

In this study, we aimed to report our experience with pediatric patients in whom airway management was established using F-LMA during adenoidectomy with or without the insertion of a ventilation tube in the eardrum during operations under general anesthesia.

## METHODS

This study was approved by the Baskent University Institutional Review Board (Project no: KA15/321) and Trial registration: Clinical Trials.gov Identifier: NCT02708043. Eligible study subjects were 1-16 years of age, ASA (American Society of Anesthesiologists) I-II, and scheduled for elective adenoidectomy with or without the insertion of a ventilation tube in eardrum under general anesthesia using F-LMA between June 2012 and November 2015. Exclusion criteria were emergency surgery, extreme obesity, and signs of a significant clinical infection. Written, informed consent was obtained from the parents or guardians of each patient.

Patient data were retrospectively reviewed from prospective chart records used in our anesthesia clinic. Eight hundred fourteen pediatric patients aged 1-16 years underwent adenoidectomy with the placement of F-LMA. The patients’ medical data were obtained from their perioperative anesthesia forms, medical files, and the medical information system used at our hospital.

The medical records of the study subjects were used to record age, weight, comorbid conditions, duration of operation, premedication status, agents used for premedication, anesthesia induction, postoperative analgesia, number and type of LMAs used, intraoperative and postoperative complications (laryngospasm, bronchospasm, breath holding, pulmonary aspiration, ventilation difficulties, nausea, and vomiting), and the need for ETI due to inadequate visual surgical field, airway leaks, or inadequate ventilation. Hemodynamic data and vital signs of the patients were assessed to determine subjects with hypotension, bradycardia, F-LMA dislocation during or after placement of a mouth gag, difficult ventilation, and desaturation. Additionally, postoperative complications during or after F-LMA removal and time to discharge were recorded. A prospective questionnaire was used to assess a surgeon’s satisfaction in cases in which an F-LMA was used.

Children with peripheral venous access were premedicated intravenously (IV) with 0.1 mg/kg midazolam, 0.5 mg/kg ketamine and 0.01 mg/kg atropine and were then taken to the operating room. In all patients 3-lead ECG, noninvasive blood pressure, peripheral oxygen saturation (SpO2), and end-tidal carbon dioxide were monitored. ETI equipment was prepared for possible inadequate ventilation. In these patients, anesthesia was induced using 3 mg/kgpropofol and 2 μg/kg fentanyl IV, and maintenance was achieved using 2% sevoflurane.

**Fig. 1:**
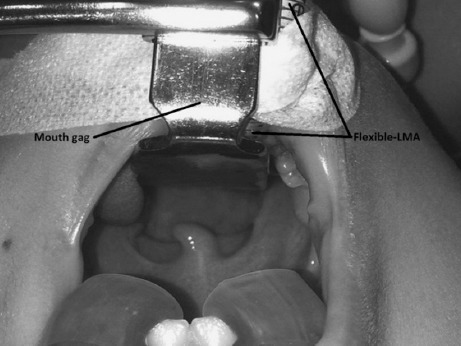
Patient image of the mouth gag with F-LMA inserted. F-LMA under the mouth gag without kink

Anesthesia induction was achieved using IV 3 mg/kgpropofol and 2 μg/kg fentanyl, and the anesthesia maintenance was achieved using 2% sevoflurane. No muscle relaxant was administered. An appropriate-sized, weight-based F-LMA with a metal spiral (reinforced) recommended by the manufacturer was placed, and its cuff was inflated not to exceed the volumes recommended by the manufacturer. Then, air or leak sounds were checked in patient’s mouth. After confirming adequate ventilation, the F-LMA was fixed at the midline. After placing an adequate mouth gag with a weight-based size, the operation began (Fig 1). The operation was terminated after the removal of the adenoid tissue by curettage, achieving hemostasis, and aspiration of the oral cavity contents.

In patients with adequate spontaneous respiratory effort at the end of the operation, the F-LMA was removed, mask ventilation initiated and then transferred to the postoperative anesthesia care unit (PACU).

All patients were closely monitored by pulse oximetry in the PACU. They were also closely followed for side effects, such as bleeding, hoarseness, sore throat, nausea, vomiting, and agitation. Patients who complained about pain were administered IV 1 mg/kg tramadol. Patients with nausea and/or vomiting were administered IV 0.1 mg/kgondansetron (max 4 mg). After evaluation using the modified Aldrete scoring system, patients with 10 points or more were sent to the ward.

The compact program SPSS 17.0 (SPSS Inc., Chicago, IL, USA) was used for statistical analysis of the data. Descriptive statistical methods were used to analyze the data. Categorical variables were expressed as numbers and percentages, whereas numerical variables were expressed as a mean and standard deviation (and as a median and minimum–maximum, when necessary).

## RESULTS

Eight hundred fourteen patients were screened for inclusion in the study. Patients’ demographics, including age, weight, gender, ASA status, duration of surgery, PACU time and comorbidities are shown in Table-I.

**Table-I:** Demographic and baseline clinical characteristics of the patients.

Age (y)	5.67 ± 2.87
Weight (kg)	23.29 ± 11.09
Gender (Female/Male)	483/331
ASA (I/II/III) (n)	655/143/16
Duration of surgery (min)	33.72 ± 14.56
PACU time (min)	17.78 ± 3.60
Co-morbidity	159 (%19.6)
Asthma	60 (%7.4)
Bronchitis	48 (%5.9)
Epilepsia	16 (%2.0)
Congenital cardiac disease	14 (%1.7)
Thalassemia	5 (%0.6)
Sickle cell disease	5 (%0.6)
Others	11 (%1.2)

Data are expressed as mean ± SD, Number of subjects (n) and percent (%), ASA (American Society of Anesthesiologists), PACU (Post Anesthesia Care Unit).

The indications for surgical interventions were snoring (40.5%), open mouth sleeping (39.8%), recurren otitis media (9.8%), recurren adenitis (5.4%) and recurren sinusitis (4.4%). Four hundred ninety-three (60.6%) patients received premedication, and 321 (39.4%) patients had no peripheral venous access and received a peripheral venous line under sedation with sevoflurane inhalation.

LMA failure occurred in 2 (0.24%) patients, and the F-LMA was replaced with ETI in these patients. Two (0.24%) patients suffered a perioperative airway complication: one being intraoperative laryngospasm and the other was laryngospasm while emerging from anesthesia. No mortalities or life-threatening morbidities occurred in any of the patients. All patients were discharged the same day.

A prospective comparison of F-LMA and ETI was performed by six otolaryngologists, and 3 (50%) of these otolaryngologists stated that there was no significant difference between the two methods, whereas the remaining three stated that F-LMA limited the surgical visual field. Five (83.3%) otolaryngologists advocated that F-LMA was associated with better patient comfort than ETI, and all otolaryngologists stated that the operation time was significantly shortened when using F-LMA and wished to continue using F-LMA in future adenoidectomy operations Table-II.

**Table-II:** Opinions of the surgeons’ regarding the use of the F-LMA in adenoidectomies.

	*1 (Better)*	*2 (Same)*	*3 (Worse)*
Quality of surgical vision	0	3 (%50)	3 (%50)
Comfort of operation	1 (16.7)	4 (%66.7)	1 (%16.7)
Postoperative patient comfort	5 (%83.3)	1 (%16.7)	0
Postoperative complications	3 (%50)	3 (%50)	0
Operation time	6 (%100)	0	0
Surgeon satisfaction	2 (%33.3)	3 (%50)	1 (%16.7)
Personal opinion	1 (%16.7)	4(%66.7)	1 (%16.7)

I want to continue use of the F-LME, n (%)	***Yes***		***No***

	6 (%100)		0

Data are expressed as number of surgeon and percent, n (%).

## DISCUSSION

In this study, we evaluated the safety of F-LMA use in pediatric patients who underwent adenoidectomies with/without ventilation tube insertion in the eardrum under general anesthesia. We found that F-LMA use in these patients resulted in fewer perioperative complications, a moderate surgical view, a short PACU time, high patient comfort during the perioperative period and high satisfaction scores for surgeons with safe airway management.

General anesthesia application with LMA is frequently preferred in brief and moderately long surgeries in children.^6^,^7^ LMA is a good alternative to ETI, especially during elective surgical applications performed on an outpatient basis. LMA use shortens the duration of the stay in the operating room, eliminates the need for muscle relaxants and, thus, the use of choline esterase inhibitors, which have multiple side effects, such as bradycardia, bronchoconstriction, and hypersalivation.^8^ Another important advantage of LMA use is that, as laryngoscopy with laryngeal and tracheal stimulation is avoided, an exaggerated hemodynamic response and a possible physiological ETI-induced response are avoided. Additionally, some previous studies have reported shorter surgical duration, more rapid recovery, and more common same-day discharge rates when using LMA, resulting in reduced overall hospital costs and greater patient satisfaction.^9^,^10^ We agree with this opinion, and we are also happy as many anesthesiologists do not to use muscle relaxants for each patient.

Some studies have reported that LMA is associated with some disadvantages in adenoidectomy^11^ or tonsillectomy.^5^ These studies have reported that the conversion from LMA to ETI was required as a result of the limited surgical visual field, air leakage around the LMA, or difficult ventilation and/or problems related to oxygenation. Various studies involving small patient groups (patient number < 150) have reported a need for conversion from LMA to ETI at a rate of 9% to 11.4%, mainly due to airway obstruction and secondary to a LMA kink or a limited surgical visual field.^5^,^12^ Another study involving 1126 patients undergoing adenoidectomies showed a rate of conversion from LMA to ETI of 0.6%.^1^ It is noteworthy that a classic LMA was used for the adenoidectomies/adenotonsillectomies in the studies mentioned above. In our study, conversely, two patients (0.2%) required a conversion from LMA to ETI. We believe that the greater failure and complication rates in previous studies may have been due to the use of a classical LMA instead of an F-LMA.^1^,^3^,^5^,^11^,^12^ Other reasons may be inadequate ventilation due to inadequate anesthesia depth, respiratory superposition, excessive air leakage due to high airway pressure, or dislocation of the LMA during the procedure.

**Fig. 2:**
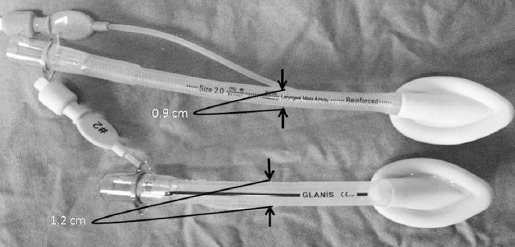
Diameter difference between the classic LMA versus F-LMA.

**Fig. 3:**
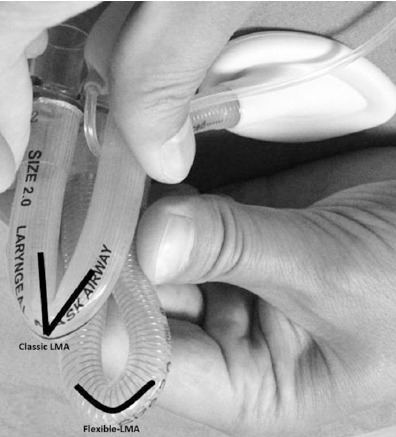
Kink difference between the classic LMA versus F-LMA.

We think that using a classic LMA is an important reason for the inadequate surgical visual field during adenoidectomies. There is a significant difference between the diameters of the classical LMA and the F-LMA (Fig. 2). The advantages of using a F-LMA during adenoidectomies include a better surgical visual field after the placement of the mouth gag and, by preventing the LMA tube from being compressed or kinked under the mouth gag (Fig. 3). As a result of these advantages, F-LMA is markedly superior over classical LMA for airway management in surgeries such as adenoidectomy and adenotonsillectomy.

When both our own experiences and the results of previous adenoidectomy studies are taken into account, we believe that a lower rate of conversion from LMA to ETI in larger studies is related to the type and diameter of LMA and the greater experience of surgeons and anesthesiologists with this procedure compared with the LMA placement technique. We preferred the use of F-LMA during adenoidectomy cases because it provides a better surgical visualization due to its smaller tube diameter, and due to its flexible structure, this device is also associated with lower rates of kink and obstruction when placing the mouth opener (Fig. 2 and 3). However, as Simon et al. reported, the placement of a F-LMA can be more difficult when an appropriate technique is not used.^13^ We also agree with this view, and we believe that this difficulty can be overcome by using an appropriate technique and with increasing experience.

Whereas limited surgical vision was not a cause for conversion to ETI in some studies 1,3,14, difficulties experienced with surgical site access were related to a conversion to ETI in some others.^15^,^16^ Although three (50%) otolaryngologists participating in this study stated that they had a more limited access and visualization of the surgical site with the use of F-LMA, they felt no need for conversion to ETI. The views of the surgical team members varied by the number of applications they performed and their experience with the method. A greater percentage of surgeons who had performed a fewer number of applications had negative views with respect to F-LMA because of being unfamiliar with F-LMA application in adenoidectomy operations, whereas a greater percentage of experienced surgeons performing more adenoidectomies had a positive attitude toward F-LMA.

Several studies on LMA and ETI during adenotonsillectomy and other surgical procedures reported a significant increase in the rate of laryngospasm with ETI compared with LMA 14-17 whereas some others failed to show any significant increase in the rate of laryngospasm with ETI.^11^ We believe that the incidence of complications associated with LMA use, include breath holding, laryngospasm, bronchospasm, increased air leakage, desaturation and ETI requirement, increase when adequate anesthesia depth cannot be achieved by volatile anesthetics due to air leaks secondary to the incomplete placement of the LMA. In our study, one patient suffered laryngospasm after anesthesia depth became lighter, and this problem was overcome by administering additional propofol. One patient in our study experienced laryngospasm that was controlled by administering 100% oxygen flush via face mask.

Hem et al.^5^ reported that the operation duration was prolonged when using LMA mainly due to more difficulty accessing the surgical site. Conversely, Ranieret al.^12^ found no significant difference between LMA and ETI with regard to the operation duration. It is noteworthy that a classic LMA was used in both of these studies. In our study, in which we used F-LMAs during adenoidectomies, patient characteristics and surgeons’ styles and operative times were variable, but all surgeons stated in our questionnaire that the duration of the operation was markedly shortened when using F-LMA. As LMA is associated with a better waking quality and postoperative patient comfort, all surgeons express positive views for using LMA in future adenoidectomies. This is a favorable advance for anesthesiologists.

Some researchers reported that using LMA during adenotonsillectomy operations in children aged three years or younger is not safe and is associated with increased complications.^18^,^19^ These authors suggested that more data from larger controlled studies are needed in children aged three years or younger. In our study, safe airway management was established by the use of F-LMA in 150 patient’s aged three years or younger undergoing adenoidectomy. We believe that the familiarity of surgeons and anesthesiologists with this method is a very important factor affecting success.

There are many clinical studies indicating better postoperative patient comfort when using LMA compared with ETI.^3^,^10^,^14^,^16^ A significantly lower rate of coughing is a striking and common finding in all these studies. In a meta-analysis of 29 randomize controlled studies, Yu et al. found that sore throat and cough were significantly more prevalent with ETI.^15^ According to the results of our study, the mean duration of the stay in the PACU was 17 minutes after LMA use, and all patients who used a LMA were discharged on the same day. Such a trend has a substantial favorable impact on patient and parent comfort and morale, healthcare costs, labor force gain, and healthcare personnel morale.

### Limitations of the study

First, our study had a retrospective design, but in fact, prospectively recorded data were assessed retrospectively. Second, the lack of an ETI group for comparison with the use of F-LMA during adenoidectomy was the main limitation of our study.

We believe that future studies with larger sample sizes comparing F-LMA and ETI may clarify this subject. We also believe that our study and clinical experience may guide clinicians in this field.

## CONCLUSION

Our study demonstrated that the use of F-LMA during the administration of general anesthesia for adenoidectomies can be performed safely with an experienced and dedicated team. Physicians should keep in mind that the use of F-LMA is a valuable option for airway safety in pediatric patients who are undergoing adenoidectomies.

### Authors’ Contribution

OO, MS conceived, designed and did statistical analysis & editing of manuscript.

OO, MS, EC, MK, AA did data collection and manuscript writing.
